# ICERA: Increasing accessibility to medical education through structured international collaboration

**DOI:** 10.1016/j.bjane.2024.844522

**Published:** 2024-05-29

**Authors:** Maxwell B. Baker, Rafael Ortega, Federico Bilotta, Jennifer Wang, Wendy Bernstein

**Affiliations:** a Boston University Chobanian & Avedisian School of Medicine, Department of Anesthesiology, Boston, USA; b Sapienza University of Rome, Policlinico Umberto I Teaching Hospital, Department of Anaesthesiology, Critical Care and Pain Medicine, Rome, Italy

*Dear Editor,*

The COVID-19 pandemic triggered sudden and significant adjustments to anesthesia residency training programs worldwide. Initially, the most pressing issues focused on concerns of reduced clinical exposure provided to trainees, largely due to the widespread cancellation of elective surgeries.^1^, ^2^, ^3^, ^4^ However, reduction of educational activities and networking opportunities, especially those across institutions, also played a large role in degrading the quality of the residency experience.^5^ Initial stages of the pandemic caused an abrupt discontinuation of in-person learning opportunities such as didactic lectures and grand rounds. With efforts to adhere to social distancing guidelines and reduction of the spread of the disease, such gatherings were temporarily paused and substituted with virtual learning modalities.^6^ Once established, the shift to virtual learning platforms became a flexible option for presenting educational material. Virtual platforms gave trainees the ability to maintain a regular attendance schedule while minimizing the risk of infection. Virtual sessions were recorded and archived, increasing the accessibility of educational material. Trainees could easily catch up on missed sessions or revisit key material at a later date, enhancing their learning experience. Furthermore, virtual platforms have fostered real-time interactions and discussions, even across geographical boundaries, regardless of where different parties are physically located.^5^

While largely beneficial, these virtual shifts brought to light an uneven access to quality virtual learning across anesthesia residency programs. This disparity was especially apparent in low- and middle-income countries, where resource limitations often hindered effective implementation.^7^ That being said, significant distinctions prevail among European nations, particularly evident in the divergent educational standards, structures and resources within residency programs.^8^ These variations contribute to inconsistent progress in developing core competencies and implementing best practices to ensure patient safety. For example, in Denmark, clinical experience and completion of the anesthesia residency training program is prioritized over the successful passing of a national written examination. In Switzerland, there is an emphasis on theoretical knowledge and the successful completion of a board certification test. Both approaches differ in ensuring optimal patient safety. The identification of such inconsistent standards in postgraduate medical education across the European Union, including Italy, Portugal, France, and Spain, suggest a larger gap in the need for a more standardized program.^9^

Recognizing these differences in didactic education and the increasing accessibility of virtual learning, a unique collaboration emerged between the faculty in the Departments of Anesthesiology at Boston University, in the United States, and Sapienza University, in Italy. Virtual platforms have allowed these physicians to eliminate the ocean of distance that otherwise served as a barrier to collaboration, fostering a seamless exchange of knowledge, ideas and expertise. The initial series of lectures targeted at advancing anesthesia education and training worldwide sparked a global initiative. Creating a global network of established anesthesiologists, they formed the International Collaboration for Education and Research in Anesthesia (ICERA).

## Impact

**Fig 1:**
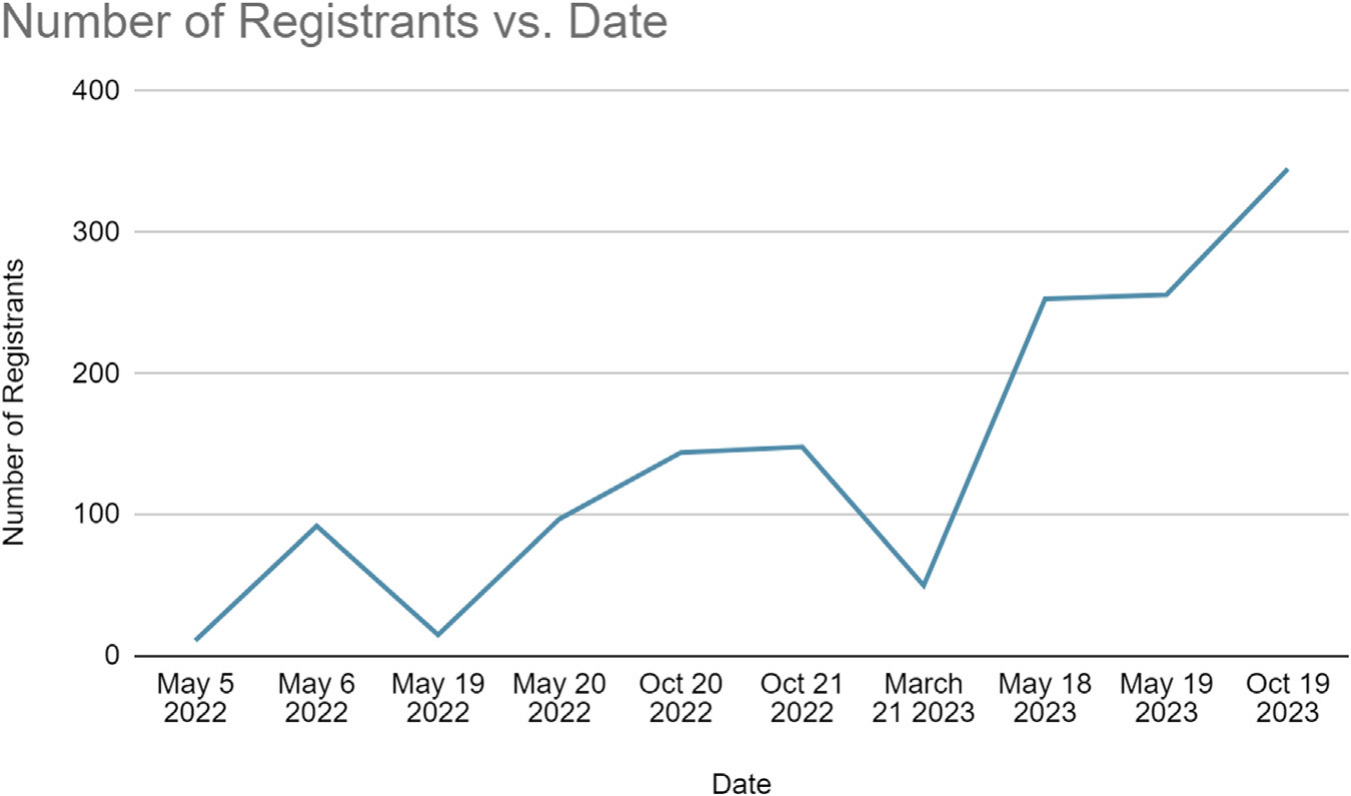
Number of registrants over time.

**Fig 2:**
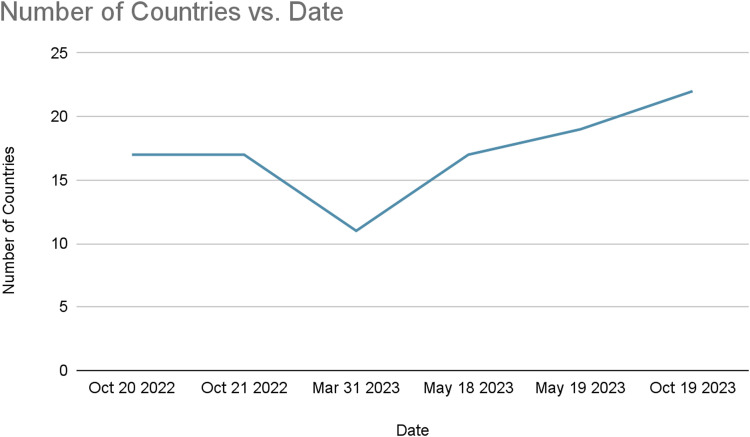
Number of countries over time. *Country data not collected before Oct 20, 2022.

From initial lectures to virtual classrooms, ICERA has morphed in a year. Spanning 20 countries and attracting over 400 participants (as shown in Table 1 and Figs. 1 and 2), ICERA has fostered a vibrant international connection between anesthesia trainees and attending physicians, solidifying its impact beyond the challenges posed by the COVID-19 pandemic.

**Table 1:** List of participating countries.

Italy	Greece	Turkey	Argentina
Montenegro	United Kingdom	Kenya	Portugal
Spain	Kosovo	Uganda	Cyprus
Greece	Albania	Romania	Brazil
Tanzania	Colombia	Switzerland	Canada
Moldova	Argentina	Poland	Germany

Empowering anesthesiology residents worldwide, ICERA provides free access to high-quality virtual courses on crucial topics. These courses, expertly designed by dedicated anesthesiologists worldwide, and vetted by ICERA's executive board, directly address the learning needs mandated by the various residency programs and stay at the forefront of global advancements. ICERA lectures are currently a freely accessible resource to the public, thus eliminating any potential financial barriers. They offer the advantage of recorded sessions accessible virtually anywhere, and with the ability to revisit key content or explore new topics at the resident's own pace.

Lectures are conducted via videoconferencing software, providing attendees real-time opportunities for interactive learning. Notably, dedicated time is allocated afterwards for further discussion, allowing trainees to directly engage with international experts outside of their institutional network. This intentional collaborative design fosters a shared understanding of emerging challenges in the ever-expanding field of anesthesia.

ICERA's collaborative reach extends beyond the classroom and the latest trends in perioperative medicine. ICERA aims to broaden the education, research, and scholarly opportunities for anesthesiology trainees worldwide. Recognizing the unique landscape of residency programs across the globe, there are opportunities to harmonize the educational framework for anesthesia didactics. This focus on standardization, particularly in regions with limited resources, holds the potential to empower future anesthesiologists and propel our field's collective expertise to new heights. Moreover, ICERA's commitment is sustained by mentoring initiatives that cater to anesthesiologists at any stage of their careers. ICERA provides a pivotal platform for participants to expand their professional networks, connecting them with experts and individuals with diverse interests beyond their immediate institutions. Additionally, the sharing of best practices in anesthesia care through ICERA contributes to a continuous enhancement in patient outcomes, reflecting a commitment to ongoing quality improvement in the field of anesthesiology.

ICERA's effects extend beyond knowledge sharing to sparking international research. Clinicians can now design multicenter studies with diverse participants, a stark contrast to the typical regional single-center projects.^10^ This shift promises not only valuable insights into regional healthcare variations, but also a groundbreaking platform for drawing broader conclusions applicable to global perioperative medicine.

ICERA keeps its global community informed with a triannual newsletter, bursting with updates, lecture highlights, and insights into evolving topics in anesthesia. The newsletter features highlights of recent and upcoming lectures, offering valuable insights into evolving topics, as well as keeping members informed of evolving best practices, with updates on consensus guidelines and anesthesia recommendations. Launched in September 2023, it is a valuable source of ongoing information for the ICERA global community.

## Conclusion

According to the World Federation of Societies of Anaesthesiologists, a critical concern within the global anesthesia community is the shortage of adequately-trained anesthesiologists in developing countries.^11^ This shortage impacts the safe and effective delivery of anesthesia services, as these are contingent on the expertise and proficiency of anesthesiologists, emphasizing the pivotal role played by comprehensive training. The situation underscores the importance of ongoing efforts to enhance access to education and mentorship in the field of anesthesia.

Addressing this challenge requires sustained international collaboration to develop effective solutions. A key aspect of this collaboration is the creation of platforms for sharing knowledge and best practices. This is where ICERA plays a significant role. ICERA offers a valuable resource for anesthesiologists at various states of their careers. ICERA provides opportunities for trainees to enhance their clinical skills and for faculty members to advance in research and professional development. This collaborative global environment is instrumental in pursuing shared goals and fostering collective, meaningful change in the field of anesthesia.

## Conflicts of interest

The authors declare no have conflicts of interest.
